# Eosinophilic granulomatosis with polyangiitis: Patient profiles from a large US allergy practice

**DOI:** 10.1016/j.jacig.2025.100437

**Published:** 2025-02-04

**Authors:** Michael E. Wechsler, Anna Kovalszki, Jared Silver, Brian Stone, William McCann, Lynn Huynh, Anamika Khanal, Mingchen Ye, Mei Sheng Duh, Arijita Deb

**Affiliations:** a National Jewish Health, Denver, Colo; b University of Michigan, Ann Arbor, Mich; c US Medical Affairs–Respiratory, GSK, Durham, NC; d Allergy Partners of Western North Carolina, Asheville, NC; e Analysis Group, Boston, Mass; f Global Real-World Evidence & Health Outcomes Research, GSK, Philadelphia, Pa

**Keywords:** EGPA, biological products, burden of disease, eosinophilia, private practice, symptoms, relapse, oral corticosteroid, retrospective study, longitudinal study

## Abstract

**Background:**

Data on the presentation and management of patients with eosinophilic granulomatosis with polyangiitis (EGPA) in private practice are limited.

**Objective:**

We sought to characterize the profiles and disease burden of patients with EGPA in a real-world private practice setting.

**Methods:**

This was a retrospective, noninterventional, longitudinal study (GSK ID: 217426) of US Allergy Partners network data. For patients with a diagnosis of EGPA, confirmed by 2 or more EGPA clinical features, index was defined as their first visit with an Allergy Partners physician (January 2007–June 2021); postindex lasted until loss of follow-up or study end (December 2021). Patient characteristics at index, physician characteristics at any time, symptoms, treatment characteristics, and clinical outcomes postindex were assessed.

**Results:**

Of 52 patients (median follow-up, 3.7 years), 75% were diagnosed with EGPA outside the Allergy Partners network. Each patient received care from a median (Q1-Q3) of 4.0 (3.0-5.0) physician specialties. Most had asthma (92%), rhinitis (75%), and sinusitis (62%) and experienced a mean ± SD of 18.1 ± 4.3 distinct self-reported symptoms. Most (85%) used oral corticosteroids, with 73% (32 of 44) on daily doses of more than 12 mg; 60% used mepolizumab. Overall, 75% of patients (39 of 52) achieved a response (improved/controlled symptoms); 46% (24 of 52) achieved controlled status after worsened, unchanged, or active symptoms, and of these 38% (9 of 24) relapsed.

**Conclusions:**

The complex private practice presentation of EGPA, with heterogeneous patient response to standard treatments, highlights a significant disease burden and continued need for optimized treatment strategies within a multidisciplinary team approach.

Eosinophilic granulomatosis with polyangiitis (EGPA) is a rare inflammatory disease with an estimated global prevalence of 2 to 38 cases per million people.^1^, ^2^, ^3^ The disease is typically characterized by systemic vasculitis of small to medium vessels, blood and tissue eosinophilia, asthma, and frequently nasal polyps, pulmonary infiltrates, and peripheral neuropathy.^4^, ^5^, ^6^, ^7^ Patients with EGPA commonly experience relapsing-remitting symptomatology,^8^, ^9^, ^10^, ^11^ with a wide range of organ system manifestations including those of the lung; ear, nose, and throat; skin; and nervous, gastrointestinal, and cardiovascular systems.^9^, ^10^, ^11^, ^12^, ^13^ EGPA progression can lead to organ injury and impairment and to life-threatening conditions.^14^, ^15^, ^16^ EGPA is associated with antineutrophil cytoplasmic antibodies (ANCAs) in up to 40% of cases, most commonly against myeloperoxidase.^9^^,^^13^^,^^17^, ^18^, ^19^

Standard treatment for EGPA is the use of oral corticosteroids (OCSs), used alone and with immunosuppressants.^4^ Management guidelines recommend minimizing OCS use due to toxicity; as long-term use is associated with cardiovascular, bone, muscle, psychiatric, and ocular complications.^4^^,^^16^^,^^20^^,^^21^ Mepolizumab, the first-in-class anti–IL-5 humanized mAb, became available to patients with EGPA in the United States in 2017,^22^^,^^23^ followed by benralizumab in 2024.^24^ By specifically targeting IL-5, mepolizumab inhibits IL-5 signaling, with downstream impacts including reductions in eosinophilic inflammation.^23^^,^^25^ Other biologics under investigation for the treatment of EGPA include reslizumab (which also targets the IL-5 pathway), omalizumab (targeting IgE), tezepelumab (targeting thymic stromal lymphopoietin), and dupilumab (targeting IL-4/IL-13 signaling).^23^^,^^25^, ^26^, ^27^, ^28^

EGPA is mainly diagnosed and managed within academic referral centers. Previous retrospective studies in France, Italy, the United Kingdom, Japan, Canada, and the United States have described the characteristics and outcomes of patients with EGPA outside of private practice or community care settings and highlighted the high comorbidity and multiple-organ involvement in EGPA.^9^, ^10^, ^11^^,^^13^ In addition, previous Japanese and US database studies and a systematic review have indicated the substantial burden of EGPA in terms of treatment use and health care resource utilization (HCRU).^1^, ^2^, ^3^^,^^29^ However, little is known about how EGPA is diagnosed and managed in private practice and whether patient presentation and burden of disease differs from that in academic centers, which is pertinent given that underrecognition of some EGPA manifestations is likely without routine specialist involvement.^30^ Moreover, understanding and raising awareness of the disease burden across multiple clinical settings may allow earlier recognition of EGPA, thus potentially shortening time to diagnosis or treatment. The Allergy Partners network contains electronic medical record (EMR) data from 2007 onwards for approximately 1.7 million patients (currently 250,000/y) and 135 physicians across 126 practice hubs in 20 states, representing a good resource to determine the characteristics and burden of EGPA in private practice.

This study used data from the Allergy Partners network to characterize the profiles and burden of disease of patients with EGPA in a real-world, nonacademic, private practice setting and to assess the extent to which they achieved clinical goals such as symptom control and response.

## Methods

### Study design and data source

**Fig 1:**
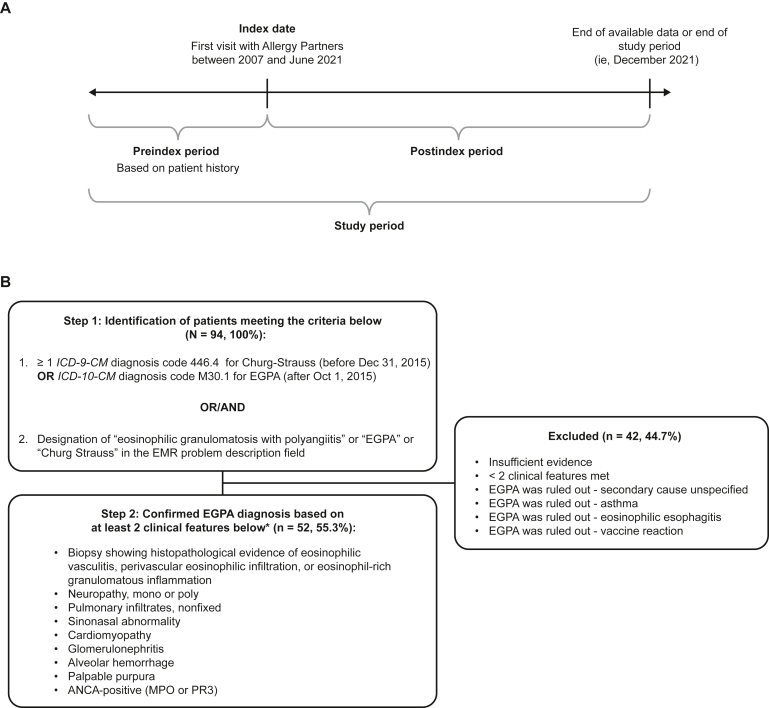
Study design (**A**) and patient identification and inclusion (**B**). ∗As per predefined EGPA selection criteria.^31^*ICD-9/10-CM, International Classification of Diseases, Ninth/Tenth Revision, Clinical Modification;MPO*, myeloperoxidase; *PR3*, proteinase 3.

This is a retrospective, noninterventional, longitudinal chart review study (GSK ID: 217426) of patients with EGPA receiving treatment through the Allergy Partners network, the largest single-specialty practice in allergy, asthma, and immunology in the United States. Data were collected from the Allergy Partners EMR, which contains structured and unstructured data, both of which were used in this study. Unstructured data (physician notes and clinical reports) underwent expert review for text relevant to identification of EGPA, disease severity, and rationale behind prescribing decisions/treatment changes. The index date was the patient’s first visit with an Allergy Partners physician between January 2007 and June 2021, regardless of whether the visit followed, coincided with, or preceded a diagnosis of EGPA. The postindex period lasted until loss to follow-up or study end (December 2021). Data reported before the first visit to the Allergy Partners physician (preindex) were based on the available patient history (Fig 1, *A*). Because no direct subject contact or primary collection of individual human subject data occurred, and study results were in tabular form with aggregate analysis that omits subject identification, informed consent was not required. On this basis, exemption from institutional review board oversight was obtained (granted by the Western Institutional Review Board, now known as the Western Institutional Review Board–Copernicus Group).

### Patient inclusion criteria

Patients with EGPA were subjected to a 2-step eligibility process.^7^ During step 1, potentially eligible patients were identified using *International Classification of Diseases, Ninth/Tenth Revision* diagnosis codes (446.4 or M30.1) or designation of “EGPA” or “Churg-Strauss” in the problem description field of their EMR (Fig 1, *B*). At step 2, the diagnosis of EGPA was confirmed on the basis of the presence of 2 or more supporting clinical features of EGPA noted in chart review by 2 expert allergists from Allergy Partners.^31^ These features are shown in detail in Fig 1, *B*.

### Characteristics and outcomes

Characteristics and outcomes assessed included patient demographic characteristics at index, physician characteristics and patient comorbidities at any time, laboratory assessments, symptoms and manifestations, treatment characteristics and outcomes, and HCRU postindex. Results generated for the postindex period included the index date.

Laboratory tests postindex included absolute blood eosinophil count (BEC), Asthma Control Test, FEV_1_, and the ANCA test. Symptoms and manifestations assessed postindex included the number of distinct patient-reported symptoms by organ class. Treatment characteristics included the proportion of patients using respiratory medications including OCSs, biologics, and immunosuppressants. OCS use was also assessed (number of prescriptions per-patient-per-year [PPPY] and prednisone-equivalent daily dose by categories of ≤6 mg/d, >6 to ≤12 mg/d, and >12 mg/d, as defined by a previous study^32^).

Postindex clinical outcomes included the proportion and number of patients achieving a treatment response, achieving controlled status and EGPA relapse. The time from index date to first response or first controlled status, and the duration of first response or first controlled status were also assessed.

Two types of response were defined: response (symptoms only), defined as physician-reported improved/controlled symptoms, and response (hematologic and symptoms), assessed when patients had a BEC assessment within 30 days of a symptom response and classified as complete or partial. A complete response was defined as physician-reported improved/controlled symptoms and normal BEC of 500 or fewer cells/μL within 30 days of each other. A partial response was defined as physician-reported improved/controlled symptoms and BEC not yet in the normal range within 30 days of response.

Controlled status was defined as physician-assessed controlled status after worsened, unchanged, or active symptoms, determined on presence of appropriate key words in the “Impression” field of the EMR (eg, “controlled,” “asymptomatic,” and “resolved”). In the absence of key words in the EMR, the History of Present Illness was reviewed. All patients reported as “controlled” were “responders.” Relapse was defined as physician-reported worsening or active symptoms with any previous assessment of controlled symptoms.

Duration of the first response (or first controlled status) was defined as the time from first response (or first controlled status) to the next relapse or end of study. HCRU was reported as number of all-cause and EGPA-related hospitalizations, emergency room visits, and outpatient visits PPPY.

### Statistical analysis

Physician and patient characteristics, in addition to outcomes, were reported using descriptive statistics, and no comparative analyses were conducted. Mean (± SD) and median (with interquartile range [IQR]) values were used to summarize continuous variables; frequencies and proportions (counts and percentages) were used for categorical variables.

## Results

In total, 94 patients met 1 or both identification criteria for EGPA, of whom 52 had an EGPA diagnosis confirmed by the presence of 2 or more supporting clinical features (Fig 1, *B*) and were included in the overall study population. The median (IQR) follow-up duration was 3.7 years (0.7-6.3).

### Physician characteristics

Half the patients were referred to the Allergy Partners network by their primary care physician (PCP), 21% by their pulmonologist, and 13% by another allergist/immunologist (Table I). Patients had a record of care from a median (IQR) of 4 (3-5) different specialists over the study period; the most represented specialties in patient care teams (after allergists/immunologists) were otolaryngologists (60%), pulmonologists (58%), and rheumatologists (56%).

**Table I:** Characteristics of referring and care physicians for patients with EGPA (structured data)

Characteristics	Overall (N = 52)
Specialty of referring physician, n (%)	
PCP	26 (50)
Pulmonologist	11 (21)
Allergist/immunologist	7 (13)
Self-referral	3 (6)
ENT physician	1 (2)
Gastroenterologist	1 (2)
Hematologist/oncologist	1 (2)
Rheumatologist	1 (2)
Unknown	1 (2)
No. of specialties in care team per patient	
Mean ± SD	3.9 ± 1.6
Median (IQR)	4.0 (3.0-5.0)
Specialty of physician in care team, n (%)	
Allergist/immunologist	52 (100)
ENT	31 (60)
Pulmonologist	30 (58)
Rheumatologist	29 (56)
Cardiologist	19 (37)
Hematologist/oncologist	11 (21)
Gastroenterologist	8 (15)
Dermatologist	6 (12)
Neurologist	6 (12)
Nephrologist	4 (8)
Infectious disease	3 (6)
Endocrinologist	2 (4)

*ENT*, Ear, nose, and throat.

### Patient demographic and clinical characteristics

Most of the patients (75%) were diagnosed with EGPA outside of the Allergy Partners network, and the mean ± SD patient age was 50.2 ± 16.9 years at diagnosis and 53.1 ± 16.4 years at index. Most patients (63%) were female, from southern states (65%), and had commercial health insurance (56%) (Table II). The most common comorbid conditions during the entire study period were diseases of the circulatory system (98%), the respiratory system (96%) (including asthma [92%] and rhinitis [75%]), and the gastrointestinal system (40%) (including gastroesophageal reflux disease [33%]) (Table III).

**Table II:** Demographic characteristics of patients with EGPA recorded either on or before the index date (structured data)

Characteristics	Overall (N = 52)
Diagnosis of EGPA received within Allergy Partners, n (%)	
Yes	12 (23)
No	39 (75)
Unknown	1 (2)
Age (y), mean ± SD	
At diagnosis	50.2 ± 16.9
At index date∗	53.1 ± 16.4
Age category (y) at index date, n (%)	
<18	3 (6)
18-34	4 (8)
35-44	8 (15)
45-54	8 (15)
55-64	14 (27)
65-74	13 (25)
≥75	2 (4)
Sex, female, n (%)	33 (63)
Calendar year of index date, n (%)	
2007-2009	7 (14)
2010-2012	9 (17)
2013-2015	9 (17)
2016-2018	16 (31)
2019-2021	11 (21)
Index date from December 2017 onwards,† n (%)	19 (36.5)
Payer type, n (%)	
Commercial	29 (56)
Medicare	21 (40)
Self-insured	1 (2)
Unknown	1 (2)
Region,‡ n (%)	
South	34 (65)
West	8 (15)
Midwest	6 (12)
Northeast	4 (8)

*FDA*, US Food and Drug Administration.

∗ Defined as first patient visit with Allergy Partners between 2007 and June 2021.

† Date of FDA approval of mepolizumab for EGPA.

‡ The region categories were based on those of US census.

**Table III:** Comorbid conditions∗ of patients with EGPA during the entire study period occurring in more than 1 patient (structured data)

Conditions	All time points (N = 52)
Disease of the circulatory system, n (%)	51 (98)
Hypertension	6 (12)
Pericardial effusion	2 (4)
Disease of the respiratory system, n (%)†	50 (96)
Asthma	48 (92)
Rhinitis	39 (75)
Sinusitis	32 (62)
COPD	8 (15)
Respiratory infection	6 (12)
Disease of the gastrointestinal system, n (%)	21 (40)
GERD	17 (33)
Disease of the nervous system, n (%)	7 (13)
Sleep apnea	2 (4)
Headache	2 (4)
Neuropathy	2 (4)
Infectious and parasitic diseases, n (%)	7 (13)
Thrush	2 (4)
Anxiety and depression, n (%)	3 (6)
Anxiety	3 (6)
Other, n (%)†	45 (87)
Cough	8 (15)
Drug allergy	8 (15)
Conjunctivitis	7 (13)
Urticaria	6 (12)
Wheezing	6 (12)

	**OCS users (N = 44)**

Comorbidity, n (%)‡	
Otolaryngologic	43 (98)
Respiratory	38 (86)
Infections	24 (55)
Gastrointestinal	17 (39)
Hematologic/oncologic	13 (30)
Dermatologic	13 (30)
Ophthalmologic	8 (18)
Cardiovascular	9 (21)
Psychiatric	5 (11)
Bone- and muscle-related	5 (11)
Metabolic	5 (11)

*COPD*, Chronic obstructive pulmonary disease; *GERD*, gastroesophageal reflux disease.

∗ Categories are not mutually exclusive.

† Includes the top 5 comorbid conditions in this category.

‡ Comorbidities were assessed among OCS users during the postindex period.

Regarding postindex laboratory assessments, 41 (79%) patients had 1 or more BEC assessments, of which 12 (29%) had a BEC more than or equal to 1500 cells/μL (median [IQR], 700 [240-1500] cells/μL). In total, 34 (65%) patients had 1 or more Asthma Control Test result, 28 of whom had a score of 20 to 25 (defined as well controlled; overall mean ± SD, 23.3 ± 3.0), and 44 (85%) had 1 or more FEV_1_ result, with a mean of 79.6% ± 18.4%. Of the 19 (37%) patients who had 1 or more ANCA screens, 4 (21%) were ANCA-positive (2 were antimyeloperoxidase-positive and 2 were anti–proteinase 3-positive) (Table IV; see also Table E1 in this article’s Online Repository at www.jaci-global.org).

**Table IV:** Laboratory test results of interest and clinical outcomes of patients with EGPA during the postindex period derived from chart review data

Outcomes Laboratory tests‡	All time points postindex	First year of care postindex∗†
	N = 52	N = 48§
BEC (cells/μL), median (IQR)	700.0 (240.0-1500.0)	700.0 (241.5-1500.0)
Eosinophil (%), median (IQR)	7.0 (3.3-16.5)	7.5 (3.5-16.0)
White blood cell count (10^3^ cells/μL), median (IQR)	9.0 (7.6-10.9)	9.1 (7.7-10.9)
ANCA screen, n (%)		
≥1 assessment	19 (37)	11 (23)
Positive result	4 (21)	2 (18)
ACT score (0-25), median (IQR)‖	25.0 (22.0-25.0)	22.5 (19.0-25.0)

**Clinical outcomes**	**N = 52**	**N = 16**

Observation period (y)		
Mean ± SD	4.1 ± 3.5	0.4 ± 0.3
Median (IQR)	3.7 (0.7-6.3)	0.2 (0.1-0.6)
Response (symptom only; physician-reported improved/controlled symptoms)		
Patients who achieved response, n (%)	39 (75)	6 (38)
Time (mo) to first response from index, median (IQR)	5.6 (1.4-21.7)	1.2 (0.0-2.6)
Duration (mo) of the first response, median (IQR)	19.5 (9.5-40.1)	4.5 (0.0-8.2)
Response (defined by hematologic and symptoms)¶		
Patients who had BEC assessment within 30 d of response, n	35	10
Patients who achieved complete response, n (%)	9 (26)	1 (10)
Time (mo) to first complete response from index, median (IQR)	5.6 (1.0-11.1)	0 (0.0-0.0)
Duration (mo) of the first complete response (time from first complete response to next relapse or end of follow-up), median (IQR)	33.1 (6.4-44.8)	1.8 (1.8-1.8)
Controlled status (physician-assessed)		
Patients who achieved controlled status (after worsened, unchanged, or active symptoms), n (%)	24 (46)	4 (25)
Time (mo) to first controlled status from index, median (IQR)	16.2 (1.2-26.5)	0.6 (0.0-1.2)
Duration (mo) of first controlled status (time from first controlled status to next relapse or end of follow-up), median (IQR)	9.7 (3.7-28.5)	4.5 (0.9-8.9)
EGPA relapse (physician-reported worsening or active symptoms)		
Patients who had previous assessment of controlled status, n	24	5
Patients who experienced relapse, n (%)	9 (38)	1 (20)
Deaths, n (%)	0 (0)	0 (0)

*ACT*, Asthma Control Test.

∗ Index date was defined as the patient’s first visit with Allergy Partners between 2007 and June 2021.

† Includes patients with 0- to 12-mo follow-up; patients with more than 12-mo follow-up were not included.

‡ Mean test values are calculated from the highest result reported values.

§ Includes all patients with at least 1 laboratory assessment during the 0- to 12-mo follow-up.

‖ ACT score defined as well controlled (well controlled: 20-25; not well controlled: 16-19; very poorly controlled: <16).

¶ Patients who did not achieve a complete response did not achieve a partial response either.

### Symptoms and clinical manifestations

**Fig 2:**
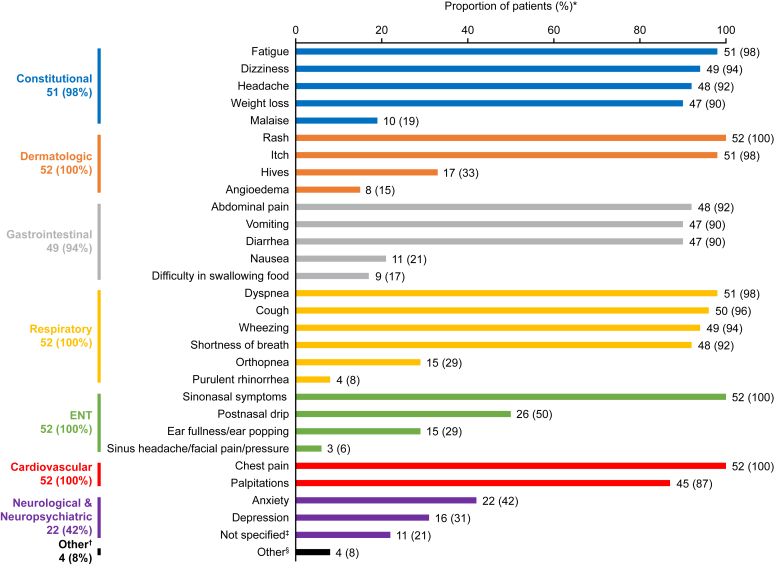
Symptoms and manifestations experienced by patients with EGPA during all time points postindex (structured and unstructured data). ∗Recorded symptoms were not necessarily specific to EGPA. †These symptoms may require review of past medical history before the index date. ‡Symptoms reported as organ-level manifestations or symptoms, including neurological symptoms and mental health symptoms. §Including infections, musculoskeletal, rheumatological, hematological, and endocrinological symptoms. *ENT*, Ear, nose, and throat.

All 52 patients self-reported at least 1 EGPA-associated symptom postindex, with a mean ± SD of 18.1 ± 4.3 distinct symptoms. The most common patient-reported constitutional symptoms were fatigue (98%), dizziness (94%), and headache (92%); dermatologic symptoms such as rash (100%) and itch (98%), sinonasal symptoms (100%), and gastrointestinal symptoms such as abdominal pain (92%), vomiting (90%), and diarrhea (90%) were also reported (Fig 2). Patient-reported respiratory and cardiovascular symptoms were also widely reported, most commonly dyspnea (98%), cough (96%), wheezing (94%), shortness of breath (92%), chest pain (100%), and palpitations (87%). Other symptoms of note included anxiety (42%), depression (31%), and weight loss (90%) (Fig 2).

### Treatment characteristics

**Fig 3:**
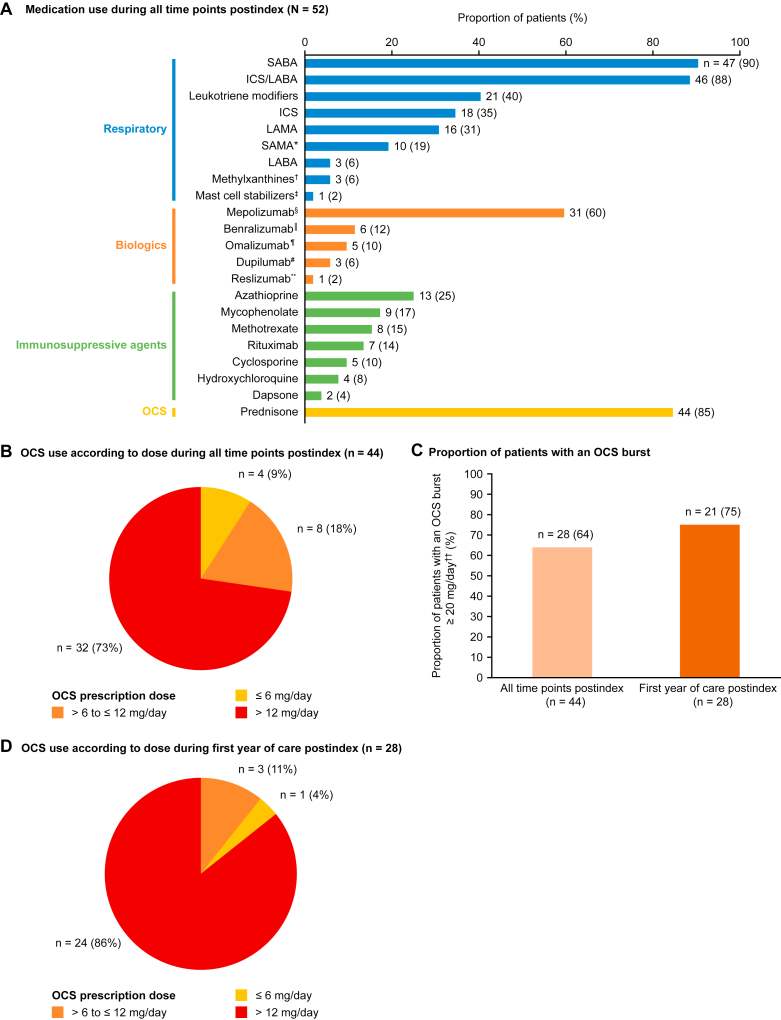
Medication use (**A**) and OCS use according to dose and burst use (**B-D**) in patients with EGPA during the postindex period (structured and unstructured data). ∗Any indication of ipratropium was flagged as SAMA. †Included theophylline, aminophylline, and dyphylline drugs. ‡Mast cell stabilizers included cromolyn and nedocromil. §The FDA first approved mepolizumab for severe asthma on November 4, 2015; ^‖^The FDA first approved benralizumab for severe eosinophilic asthma on November 14, 2017. ¶The FDA first approved omalizumab for moderate to severe persistent asthma on June 20, 2003. #The FDA first approved dupilumab for moderate to severe asthma on October 19, 2018. ∗∗The FDA first approved reslizumab for severe asthma on March 23, 2016. ††OCS bursts were defined as a prescription for OCS with 2 to 28 days of supply and an average daily dose equivalent to 20 mg or higher prednisone. *FDA,* US Food and Drug Administration; *ICS,* inhaled corticosteroid; *LABA,* long-acting β_2_-agonist; *LAMA*, long-acting muscarinic antagonist; *SABA*, short-acting β_2_-agonist; *SAMA*, short-acting muscarinic antagonist.

The most commonly used respiratory medications during the postindex period were short-acting β_2_-agonist (90%) and inhaled corticosteroid/long-acting β_2_-agonist (88%). Immunosuppressive agents were used, the most common including azathioprine (25%), mycophenolate (17%), and methotrexate (15%) (Fig 3, *A*). The most used biologic was mepolizumab (n = 31 [60%]). Of the 31 patients treated with mepolizumab, 22 patients received a dose of 300 mg at least once (the approved dose for hypereosinophilic syndrome and EGPA), whereas 24 patients were prescribed other doses at least once. Benralizumab (n = 6 [12%]) and omalizumab (n = 5 [10%]) were the next most commonly used biologics after mepolizumab (Fig 3, *A*). In total, 44 (85%) patients had been prescribed prednisone at least once postindex, of whom 32 (73%) received a dosage of more than 12 mg/d (Fig 3, *B*) and 28 (64%) had an OCS burst (2-28 days of supply and an average daily dose equivalent to ≥20 mg prednisone; Fig 3, *C*). The lowest daily OCS dose recorded over the study period was 1 mg/d, and the highest was 60 mg/d. The median (IQR) number of OCS prescriptions was 1.5 PPPY (0.8-5.9). Of the 28 patients with 1 or more OCS prescriptions within the first 12 months postindex, 24 (86%) were using a high dosage (Fig 3, *D*) and 21 (75%) had an OCS burst (Fig 3, *C*). Among these 28 patients, the median (IQR) number of OCS prescriptions was 0.5 PPPY (0.2-4.2).

### Clinical outcomes

A response, defined as physician-reported improved/controlled symptoms (without hematological markers), was achieved by 39 (75%) patients postindex. The median (IQR) time to first response from index was 5.6 months (1.4-21.7), and the median (IQR) duration of the first response was 19.5 months (9.5-40.1) (Table IV). Of note, 27 (69%) responders were treated with biologics (25 [64%] with mepolizumab) and 34 (87%) with OCSs (see Table E2 in this article’s Online Repository at www.jaci-global.org). Among the 35 patients with hematological markers (BEC assessment) within 30 days of response, 9 (26%) achieved a complete response (Table IV). The median (IQR) time from index to first response was 5.6 months (1.0-11.1), and the median (IQR) duration of the first response was 33.1 months (6.4-44.8). Here, 5 (56%) of the responders were treated with biologics (mepolizumab exclusively) and 8 (89%) with OCSs (Table E2). Of all 52 patients, 24 (46%) achieved controlled status, at a median (IQR) time of 16.2 months (1.2-26.5) and for a median (IQR) duration of 9.7 months (3.7-28.5). Among these 24 patients, 9 (38%) experienced a relapse. No patient had a record of death during the postindex period (Table IV).

### All-cause and EGPA-specific HCRU

Postindex, 8 (15%) patients had 1 or more all-cause hospitalizations, with a mean ± SD of 0.5 ± 0.7 hospitalizations PPPY, and 4.6 ± 3.6 days was the duration of stay per hospitalization (Table V). Two (4%) patients had all-cause emergency room visits, with a mean number of visits of 0.1 ± 0.0 PPPY. All 52 patients required an all-cause outpatient visit, with a mean number of visits of 11.1 ± 10.6 PPPY. EGPA-related HCRU was similar to all-cause HCRU (Table V).

**Table V:** HCRU during the entire postindex period and 0 to 12 months postindex

HCRU	All time points postindex (N = 52)	First year of care postindex (N = 52)
*All-cause*		
Hospitalizations		
Patients with ≥1 hospitalization, n (%)	8 (15)	1 (2)
No. of hospitalizations, (PPPY∗), mean ± SD	0.5 ± 0.7	2.1 ± 0.0
Duration (d) of stay per hospitalization, mean ± SD	4.6 ± 3.6	—
Emergency room visits		
Patients with ≥1 visit, n (%)	2 (4)	1 (2)
No. of visits (PPPY∗), mean ± SD	0.1 ± 0.0	0.1 ± 0.0
Outpatient visits†		
Patients with ≥1 visit, n (%)	52 (100)	52 (100)
No. of visits (PPPY∗), mean ± SD	11.1 ± 10.6	7.3 ± 11.2
*EGPA-related*‡		
Hospitalizations		
Patients with ≥1 hospitalization, n (%)	8 (15)	1 (2)
No. of hospitalizations (PPPY∗), mean ± SD	0.5 ± 0.7	2.1 ± 0.0
Duration (d) of stay per hospitalization, mean ± SD	4.6 ± 3.6	—
Emergency room visits		
Patients with ≥1 visit, n (%)	1 (2)	0 (0)
No. of visits (PPPY), mean ± SD	0.1 ± 0.0	—
Outpatient visits†§		
Patients with ≥1 visit, n (%)	52 (100)	50 (96)
No. of visits (PPPY), mean ± SD	7.8 ± 6.7	5.4 ± 7.4

*COPD*, Chronic obstructive pulmonary disease; *ICD-9/10*, *International Classification of Diseases, Ninth/Tenth Revision*.

∗ Patients with ≥1 visit.

† All visits reported were provider visits and did not include visits for treatment injections (eg, mepolizumab injection) only.

‡ With an *ICD* diagnosis code for EGPA (*ICD-9*: 446.4; *ICD-10*: M30.1), asthma (*ICD-9*: 493; *ICD-10*: J45), or sinusitis (*ICD-9*: 473; *ICD-10*: J32).

§ Outpatient visit of 1 patient who was diagnosed with COPD and vasculitis was classified as an EGPA-related outpatient visit.

## Discussion

This retrospective study of longitudinal data based on structured EMR and chart reviews from a large network of allergy practices in the United States is the first to assess the characteristics and burden of EGPA in patients managed in private practices. The results indicated that patients with EGPA in private practice were treated by multiple physician specialties and experienced various symptoms and manifestations. Treatment included extensive use of OCSs and biologics. Most patients experienced improvements in symptoms, although some patients remained uncontrolled and prone to relapse, requiring hospitalization in some cases. Taken together with the results of previous retrospective studies of patients with EGPA in academic settings,^9^, ^10^, ^11^^,^^13^ patients with EGPA experience high disease burden irrespective of clinical setting. Coordinated multispecialty care alongside shared decision making is essential, but may be more challenging in a community setting versus a contained health care system.

Most of the 52 patients with EGPA were from southern states, which reflected distribution of the Allergy Partners network. Three-quarters of patients were diagnosed with EGPA outside of the Allergy Partners network. Although PCPs made half of all referrals, EGPA diagnosis may have been made by another specialist, such as a pulmonologist or a rheumatologist, before referral to the allergist. This accords with the finding of a previous study that patients with a rare disease in the United States were more than 50% more likely than patients without a rare disease to be referred to another provider.^33^ In the present study, patients with EGPA received care from an average of 4 different specialists, reflecting the heterogeneity and multiple-organ involvement of EGPA.^9^, ^10^, ^11^, ^12^, ^13^ This highlights the key role of PCPs in coordinating a multidisciplinary treatment approach. Alternatively, patients with EGPA commonly experience delays in diagnosis, possibly seeing multiple physicians before receiving a diagnosis. Shortening delays to diagnosis is crucial to allow patients to receive earlier treatment and potentially mitigate the ongoing risk of relapse and organ damage, which are key goals of EGPA therapy.^4^^,^^34^

As expected,^7^^,^^9^^,^^10^^,^^13^ diseases of the respiratory system were prevalent comorbidities observed in almost all patients (96%) at some point in the study period. Rates of anxiety (42%) and depression (31%) were overall similar to rates of anxiety and/or depression (34%) observed in a previous study in patients with EGPA.^35^ The proportion of patients with comorbid neuropathy was lower in this study’s population (4%) than in previous reports (13%-51%),^2^^,^^3^^,^^9^^,^^10^^,^^13^ despite being one of the clinical features indicating EGPA in this study and recent classification criteria.^7^ This might be due to underreporting, because neuropathy as a symptom of EGPA was not captured in the structured data in the EMR. Similarly, only a small number of patients with 1 or more ANCA tests were positive (21%), lower than the 22% to 60% of patients previously reported^7^^,^^9^^,^^13^^,^^17^; however, only just more than one-third of patients were tested for ANCA postindex. Moreover, 29% of patients had hypereosinophilia (BEC ≥ 1500 cells/μL) and 3 had confirmed diagnoses of both hypereosinophilic syndrome and EGPA, highlighting the difficulty encountered in real-world practice in differentiating between these diseases.^7^^,^^36^^,^^37^ This is particularly the case when patients demonstrate limited vasculitis manifestations and no detectable ANCA.^36^^,^^38^

Symptoms and manifestations were observed postindex across multiple organ systems. Patients experienced 18 different symptoms and manifestations on average, with some dermatologic, gastrointestinal, respiratory, cardiovascular, and constitutional symptoms reported by nearly all. However, symptoms of rash (100%) and some gastrointestinal symptoms (94%) were unexpectedly high, with previous estimates suggesting that approximately 10% and 3% to 25% of patients experience these symptoms.^9^, ^10^, ^11^^,^^13^ This difference may be due to the self-reported nature of these results, because symptoms were included if they were reported by the patient at any time during the study period, and may therefore be related to health care visits for reasons other than EGPA care.

Over the course of care in the Allergy Partners network, OCS use was high (85% of patients) and more than two-thirds of patients required daily doses of more than 12 mg. Prescriptions PPPY were higher in the overall population than among patients prescribed OCSs within 1 year or less of follow-up, indicating that OCS burden may increase over time. Importantly, both acute and long-term OCS use are associated with significant toxicity, including infections, and gastrointestinal, cardiovascular, bone and muscle, psychiatric, and ocular complications.^2^^,^^16^^,^^20^^,^^39^ In ANCA-associated vasculitides in particular, every 12 months of OCS use is associated with a 26% increased risk of organ damage (which might comprise a combination of disease- and OCS-related damage), with a 3-fold increased risk for patients still using OCSs after a mean of 7.3 years of long-term follow-up.^16^ Management guidelines and consensus recommendations propose minimizing OCS use to the lowest effective dose.^4^^,^^34^^,^^40^

The American College of Rheumatology/Vasculitis Foundation,^4^ The European Alliance of Associations for Rheumatology,^34^ and other evidence-based guidelines^41^ now recommend the use of the add-on biologic therapy, mepolizumab for remission induction in nonsevere disease (without active organ- or life-threatening disease), maintenance of remission in severe or nonsevere disease, and relapse treatment in cases without severe disease manifestations. Despite these recommendations and the OCS-sparing properties of mepolizumab,^31^^,^^42^^,^^43^ only about 60% of patients with EGPA received mepolizumab in this study. This may be due to mepolizumab first being approved in the United States for EGPA in 2017, whereas the Allergy Partners data set begins in 2007, potentially leading to an underestimation of current usage.

Although there is still potential to increase the use of OCS-sparing agents, such as mepolizumab, in EGPA management, mepolizumab treatment appeared more frequently overall in this study than in past studies of EGPA treatment in predominantly academic settings,^13^^,^^35^ possibly because of this patient population having less severe EGPA. The lack of validated disease severity indexes such as the Five Factor Score or the Birmingham Vasculitis Activity Score^44^^,^^45^ in the Allergy Partners’ EMR means that disease severity of this population cannot be confirmed. It also indicates that these tools are underused in the management of patients with EGPA in community practice versus academic settings. Patients who qualify for mepolizumab treatment may be more likely to be referred to a private allergy practice, or allergy practices may be more likely to prescribe mepolizumab.

Three-quarters of patients achieved a symptom-only response, and only one-quarter of patients achieved complete symptom and hematologic response. Notably, complete hematologic and symptom responses lasted 1.5-fold longer than those defined by symptoms only, suggesting more durable responses in patients with marked eosinophil reductions. OCS use (given as standard of care) was high in both categories of responders (∼ 90%), compared with an overall lower use of biologics (∼ 60% given as an add-on therapy). In addition, less than half the patients with EGPA achieved controlled status, and one-third of patients experienced a relapse. Furthermore, although only 15% of patients were hospitalized, all patients required frequent outpatient visits. This hospitalization incidence is lower than previous estimates (27%-31%) in the 12 months after initial EGPA diagnosis.^3^^,^^29^ This may reflect patients in these previous studies having more active disease before remission induction than in the present study in which patients may have been already diagnosed with EGPA before index. Together, these data indicate that current highly OCS-reliant standard treatment is not adequate. In clinical trials not all patients with EGPA treated with biologics achieved remission,^31^ although most demonstrated clinical benefit.^46^

There are several limitations to be considered when interpreting the results of the present study. The classification criteria used in this study were based on the MIRRA trial criteria (updated for application to a real-world study). Since the end of the study period in 2021, updated classification criteria for EGPA including the 2022 American College of Rheumatology/European Alliance of Associations for Rheumatology classification criteria have been published. Some patients diagnosed with EGPA before their visit to Allergy Partners may not have had complete diagnostic data available, meaning it is not possible to confirm how many of the patients included would meet the most recent classification criteria for EGPA. The use of some treatments was lower than anticipated and may be due to treatments prescribed by physicians outside of the Allergy Partners network not being captured. Consequently, therapies captured are likely to represent maintenance, rather than induction, therapies. In addition, the timing of treatments relative to clinical outcomes was not assessed in this study. Although all patients with EGPA would be expected to have undergone eosinophil testing, only about three-quarters of patients had a recorded test. Similarly, laboratory tests conducted outside the Allergy Partners network may not have been captured, particularly in cases in which patients did not receive most of their care from Allergy Partners; alternatively, this could be due to incomplete data entry. Symptoms were not recorded as specific to EGPA, and so some may have been overreported; conversely, because comorbidity data were generated from structured data rather than chart review, vasculitis was not captured in *International Classification of Diseases* codes. All-cause hospitalizations may be underreported because clinicians may not document other unrelated hospitalizations or instances wherein a patient was referred to other tertiary centers. Finally, the data collected did not capture whether patients were previously nor concurrently managed at academic centers. A future study direction would be to look at whether similar outcomes were achieved for patients in private practices compared with those in academic settings.

### Conclusion

This study highlights the challenges of EGPA management within the private practice setting and the need for multispecialty care. EGPA continues to represent a substantial burden to patients and the health care system in the United States. The risk of toxicity associated with prolonged exposure to standard treatments as well as the subsequent heterogeneous patient response, with some remaining uncontrolled and prone to relapse, highlight the continued need for earlier disease recognition and management as well as optimized and individual treatment strategies within a coordinated multidisciplinary team approach.

**Key messages**

• **Previous studies in an academic care setting and database studies have highlighted the heterogeneous disease characteristics and burden of disease of patients with EGPA.**
• **This is the first study to assess the characteristics and burden of patients with EGPA outside of academic referral centers. Results reveal complex patient journeys, marked by a significant disease burden featuring high comorbidity and numerous symptoms, with heterogeneous response to standard treatments, and underused targeted therapies.**
• **Understanding the disease burden across multiple clinical settings will allow the opportunity for earlier recognition of disease, potentially shortening delays to diagnosis and allowing earlier involvement of multidisciplinary team support, while also mitigating ongoing risk of relapse and organ damage. Moreover, tailored treatment plans, OCS-sparing strategies, and a focus on earlier biologic use will also alleviate treatment burden for these patients.**

## Disclosure statement

This study was funded by 10.13039/100004330GSK (GSK; ID: 217426). The sponsor was involved in the study design and implementation as well as data collection, analysis, and interpretation, writing the study report, and reviewing this article. The sponsor did not place any restrictions on access to data or statements made in the article. All authors had full access to the data on request and had final responsibility for the decision to submit for publication.

Disclosure of potential conflict of interest: M. E. Wechsler has received research funding from the 10.13039/100000060National Institute of Allergy and Infectious Diseases, the 10.13039/100000050National Heart, Lung, and Blood Institute, GSK, Sanofi, Regeneron, AstraZeneca, Upstream Bio, and Rapt Therapeutics; is a consultant for Allakos, Amgen, Areteia Therapeutics, Arrowhead Pharmaceutical, AstraZeneca, Avalo Therapeutics, Celldex, Connect Biopharma, Eli Lilly, Equillium, GSK, Incyte, Kinaset, Kymera, Merck, MyBiometry, Pharming, Phylaxis, Pulmatrix, Rapt Therapeutics, Recludix Pharma, Regeneron, Roche/Genentech, Sanofi/Genzyme, Sound Biologics, Tetherex Pharmaceuticals, Uniquity Bio, Upstream Bio, Verona Pharma, and Zurabio; has received speaker fees from AstraZeneca, GSK, Amgen, Sanofi, and Regeneron; and has served on Data Safety Monitoring Board(s) for Sentien. A. Kovalszki has received research funds from Blueprint Medicines and AstraZeneca; is part of a hypereosinophilic syndrome trial with AstraZeneca; is a consultant for GSK, ALK, and the University of Michigan Inhale Collaborative Quality Initiative, sponsored by the Blue Cross Blue Shield of Michigan; and has received honoraria as the topic editor at DynaMed for Eosinophilia: Approach to the Patient. J. Silver was employed by GSK at the time of the study; holds financial equities in GSK; and is currently employed by Amgen and holds financial equities in Amgen. B. Stone is an employee of Allergy Partners, a consulting company that received payment from GSK to conduct this study (Allergy Partners is a registered trademark); and received consulting fees from the Analysis Group for this study. W. McCann is also an employee of Allergy Partners; has received consulting fees from Aimmune, Regeneron, ARS Pharma, and AstraZeneca; and has received honoraria as a speaker for Amgen, AstraZeneca, and Regeneron. L. Huynh and M. S. Duh are employees of Analysis Group and A. Khanal and M. Ye were employees of Analysis Group at the time of the study. Analysis Group, a consulting company, received payment from GSK to conduct this study and has received research funds for previous studies from GSK, AbbVie, Apellis, AstraZeneca, Ayala Pharmaceuticals, Bayer, Blueprint Medicines, Humacyte, Janssen, Merck, Novartis, Pfizer, Sanofi, and Takeda. A. Deb is employed by GSK and holds financial equities in GSK.
