# Supplementary material for: Eosinophilic granulomatosis with polyangiitis: Patient profiles from a large US allergy practice
Source: J Allergy Clin Immunol Glob. 2025 Feb 4;4(2):100437. doi: 10.1016/j.jacig.2025.100437 (PMC11928799; doi:10.1016/j.jacig.2025.100437)
Supplement: Supplementary Tables [file mmc1.docx]

## Online Repository

### **Table EI.** Laboratory tests and clinical assessments (post-index; structured and unstructured data)

| **Laboratory tests  (post-index)*^,^** ^†^ | **All time points**  **(N=52)** | **First year of care from index visit**  **(N=48**^‡^**)** |
| --- | --- | --- |
| **BEC** |  |  |
| ≥1 assessment, n (%) | 41 (78.8) | 23 (47.9) |
| Results, cells/µL |  |  |
| Median (IQR) | 700.0 (240.0, 1500.0) | 700.0 (241.5, 1500.0) |
| 150–500, n (%) | 9 (22.0) | 7 (30.4) |
| 500–1500, n (%) | 13 (31.7) | 8 (34.8) |
| 1500–5000, n (%) | 7 (17.1) | 4 (17.4) |
| ≥5000, n (%) | 5 (12.2) | 2 (8.7) |
| **Eosinophil percent** |  |  |
| ≥1 assessment, n (%) | 36 (69.2) | 22 (45.8) |
| Results, % |  |  |
| Median (IQR) | 7.0 (3.3, 16.5) | 7.5 (3.5, 16.0) |
| **White blood cell counts** |  |  |
| ≥1 assessment, n (%) | 37 (71.2) | 22 (45.8) |
| Results, 10^3^ cells/µL |  |  |
| Median (IQR) | 9.0 (7.6, 10.9) | 9.1 (7.7, 10.9) |
| **ANCA screen**^§^ |  |  |
| ≥1 assessment, n (%) | 19 (36.5) | 11 (22.9) |
| Positive result | 4 (21.1) | 2 (18.2) |
| **Assessments  (post index)*^,^** ^†^ | **All time points**  **(N=52)** | **First year of care from index visit**  **(N=39**^‡^**)** |
| **FEV_1_ % predicted** |  |  |
| ≥1 assessment, n (%) | 44 (84.6) | 42 (87.5) |
| Results, % |  |  |
| Mean (SD) | 79.6 (18.4) | 73.0 (20.4) |
| **ACT** |  |  |
| ≥1 assessment, n (%) | 34 (65.4) | 20 (41.7) |
| Results (0–25) |  |  |
| Mean (SD) score | 23.3 (3.0) | 20.9 (5.2) |
| **Chest X-ray** |  |  |
| ≥1 assessment, n (%) | 16 (30.8) | 9 (18.8) |
| ≥1 positive result,  n (%) | 8 (50.0) | 3 (33.3) |
| ≥1 negative result,  n (%) | 8 (50.0) | 6 (66.7) |
| **Chest CT** |  |  |
| ≥1 assessment, n (%) | 15 (28.8) | 3 (6.3) |
| ≥1 finding with opacities, n (%) | 1 (6.7) | 1 (33.3) |
| ≥1 finding with infiltrates, n (%) | 2 (13.3) | 0 (0.0) |
| ≥1 finding with nodules, n (%) | 4 (26.7) | 1 (33.3) |
| **Echocardiogram** |  |  |
| ≥1 assessment, n (%) | 1 (1.9) | 1 (2.1) |
| ≥1 positive result,  n (%) | 0 (0.0) | 0 (0.0) |

*Mean test values are calculated from highest result reported values; ^†^index date was defined as the patient's first visit with Allergy Partners between 2007 and June 2021; ^‡^includes all patients with at least one laboratory assessment during the 0–12 months follow-up; ^§^“MPO”, “M-ANCA”, “P-ANCA” and “ATYP P-ANCA” were classified as MPO; “PR3” and “C-ANCA” were classified as PR3. Besides MPO and PR3. “ANCA” and “ANCA STATUS” were classified as ANCA screen.

*ACT,* Asthma Control Test*; ANCA*, antineutrophil cytoplasmic antibodies; *BEC*, blood eosinophil count*; CT*, computed tomography; *FEV_1_*, forced expiratory volume in 1 second; *IQR*, interquartile range; *MPO*, myeloperoxidase; *PR3*, proteinase-3; *SD*, standard deviation.

### **Table EII.** Detail of treatment(s) received by patients with EGPA who were labeled as responders during the post-index period

|  | **All time points  post-index (N=52)** |
| --- | --- |
| **Response (symptom only; physician-reported improved or controlled symptoms)** |  |
| Patients who achieved response, n (%) | 39 (75) |
| Responders who received biologics, n (%) | 27 (69) |
| Mepolizumab***,** n (%) | 25 (64) |
| Benralizumab**^†^,** n (%) | 5 (13) |
| Omalizumab**^‡^**, n (%) | 5 (13) |
| Dupilumab^§^, n (%) | 2 (5) |
| Reslizumab^¶^, n (%) | 1 (3) |
| Responders on OCS, n (%) | 34 (87) |
| **Response (defined by hematologic and symptoms)**** **, n (%)** |  |
| **Patients who had BEC assessment within 30 days of response** | **N=35** |
| Patients who achieved complete response**, n (%) | 9 (26) |
| Responders who received biologics, n (%) | 5 (56) |
| Mepolizumab***,** n (%) | 5 (56) |
| Benralizumab**^†^,** n (%) | 1 (11) |
| Omalizumab**^‡^**, n (%) | 0 (0) |
| Dupilumab^§^, n (%) | 1 (11) |
| Reslizumab^¶^, n (%) | 0 (0) |
| Responders on OCS, n (%) | 8 (89) |

*FDA first approved mepolizumab (for severe asthma on November 4, 2015; **^†^**FDA first approved benralizumab for severe eosinophilic asthma on November 14, 2017; **^‡^**FDA first approved omalizumab for moderate-to-severe persistent asthma on June 20, 2003; ^§^FDA first approved dupilumab for moderate-to-severe asthma on October 19, 2018; ^¶^FDA first approved reslizumab for severe asthma on March 23, 2016; ******A complete response was defined as physician-reported improved/controlled symptoms and normal BEC ≤500 cells/µL within 30 days of each other. A partial response was defined as physician-reported improved/controlled symptoms and BEC not yet in the normal range within 30 days of response. Patients who did not achieve a complete response did not achieve a partial response either.

*BEC*, blood eosinophil count; *EGPA*, eosinophilic granulomatosis with polyangiitis; *FDA*, US Food and Drug Administration; *OCS*, oral corticosteroid.
